# The effectiveness of integrated online health-coaching on physical activity and excessive gestational weight gain: a prospective randomized-controlled trial

**DOI:** 10.1007/s00404-023-07296-y

**Published:** 2024-01-13

**Authors:** Julia Téoule, Christian Woll, Jana Ray, Marc Sütterlin, Barbara Filsinger

**Affiliations:** 1 Department of Gynecology and Obstetrics, University Medical Center Mannheim, Medical Faculty Mannheim, Heidelberg University, Heidelberg, Germany; 2 Department of Psychology, Clinical Psychology of Childhood and Adolescence and Counselling Psychology, Ludwig-Maximilians-University, Munich, Germany; 3 Institute of Clinical Psychology and Psychotherapy, Clinical Psychology of Children and Adolescents, Technical University Dresden, Dresden, Germany; 4 Department of Psychology, School of Social Sciences, University of Mannheim, Mannheim, Germany

**Keywords:** Pregnancy, Fitness tracker, Device-measured, Steps

## Abstract

**Purpose:**

Low levels of physical activity during pregnancy go along with increased risks for numerous health complications. We investigated whether an integrated lifestyle intervention leads to higher levels of physical activity and reduces the rate of excessive gestational weight gain (EGWG).

**Methods:**

We conducted a randomized-controlled trial on 97 pregnant women, randomly assigned to receive an additional telehealth lifestyle intervention (experimental group, EG; *n* = 49) or conventional antenatal care (control group, CG; *n* = 48). The core lifestyle intervention comprised regular video calls, providing integrated personal support and motivation to physical activity. The primary outcome was change in physical activity measured in steps per day. An additional exploratory outcome was the proportion of participants with EGWG.

**Results:**

The mean step count during the third trimester was 6483 steps/day (EG) and 5957 steps/day (CG), respectively (*p* = 0.078). Repeated-measures ANOVA revealed a significant interaction effect (*p* = 0.045) reflecting an overall increase of 497 steps per day in the EG vs. a decrease of 300 steps per day in the CG. The proportion of participants who met the IOM recommendation for total weight gain during pregnancy was significantly higher in the EG (*p* = 0.048) and the ratio of women that gained excessively was higher in the CG (*p* = 0.026).

**Conclusions:**

We assume that the personalized online intervention supports women in increasing or at least maintaining their level of physical activity during the course of pregnancy. Additionally, it reduces the rate of excessive weight gain.

## What does this study add to the clinical work?

**Table:** 

The present analysis suggests that integrated online intervention supports women in either increasing or maintaining their level of physical activity during pregnancy. We assume that the conventional prenatal care could be supplemented with personalized telehealth intervention to promote lifestyle changes fitting to the individual needs of each woman.	

## Introduction

There is well-known evidence of the numerous benefits of regular exercise during pregnancy. The positive effects apply for both, the pregnant women and the fetus, and include—among others—decreased incidence of gestational diabetes mellitus, hypertensive disorders, macrosomia, and postpartum depression [1–4]. In addition, physical activity is an effective way to prevent excessive gestational weight gain (EGWG) [5], being associated with long-term noncommunicable diseases and epigenetic consequences for future generations [6–10]. Furthermore, EGWG increases the risk of macrosomia and cesarean section, particularly in normal weight women [11, 12]. For this reason, the Royal College of Obstetricians and Gynaecologists (RCOG) and the American College of Obstetricians and Gynecologists (ACOG) recommend that women who were not active before pregnancy engage in a moderate amount of physical activity building up to 150 min per week. Women who begin their pregnancy with a healthy lifestyle should be encouraged to continue their healthy habits [13, 14]. Despite the acknowledged benefits of physical activity, only a low proportion of women meet the current guidelines [15–17]. Even in physically active women, the activity level typically decreases in the final weeks of pregnancy [18]. Maintaining a healthy lifestyle, and thus, the activity level during pregnancy appears to be influenced on multiple levels. Nevertheless, some main barriers were identified including lack of knowledge about the risks of overweight, lack of knowledge about the recommendations, and insufficient support from healthcare professionals [19]. It is, therefore, of great importance to establish prenatal care programs that provide interactive and personalized support, clear and reliable sources of information, and improved discussion with healthcare professionals. At the same time, behavioral interventions offer a valuable approach to lifestyle changes, which is why interventions targeting physical activity or diet during pregnancy have been studied broadly [6]. However, the nature of the interventions varies widely, with some studies evaluating exclusively digital solutions, while others focus on personalized exercise programs [5, 20]. Digital health interventions are known for their numerous advantages, such as requiring fewer resources, being more cost-effective, and having greater reachability. So far, exclusively digital health interventions without any personal support have found little evidence in promoting physical activity during pregnancy [20, 21]. As such, interventions aimed at cultivating healthy lifestyles and behaviors that positively impact future generations require a complex framework that includes personal contact with a health professional and takes into account the intricate interplay between individual characteristics and social determinants.

Therefore, the objective of this randomized-controlled trial was to evaluate effectiveness of an application-based integrated lifestyle intervention and prenatal care program, named “Healthy Birth”, which provides individualized personal support from healthcare professionals, gives personalized first-hand information, and involves psychosocial factors to enhance physical activity during pregnancy. To our knowledge, no previous trials have investigated this specific type of integrated personal intervention paired with digital technology. The primary outcome was change in physical activity measured in steps per day. An additional exploratory outcome was the proportion of participants with EGWG.

## Research design and methods

The presented randomized-controlled trial (RCT), the “Healthy Birth Study”, takes a comprehensive approach to prenatal care and is designed to promote the physiological processes of pregnancy and birth. Core elements of the study aim to provide a patient-centered care and focus on biopsychosocial well-being of women and families. The Healthy Birth program supplements the conventional prenatal obstetric care in Germany, which is mainly concentrated on somatic aspects. The present analysis deals with biological outcomes and evaluated fitness tracker data. The overall goal of the intervention was to increase daily steps on individual level and to maintain the level of activity also during the third trimester of pregnancy. Further results, such as psychological and social outcomes, will be reported elsewhere.

The Healthy Birth Study was conducted at the University Medical Centre Mannheim, Germany. Participants’ enrollment began January 2022 and is currently ongoing. Results collected up to the end of January 2023 were considered for the present analysis.

The research protocol was reviewed and approved by the University of Heidelberg ethics review board II (2021-663-AF 5). The study was financially supported by the Ministry of Science, Research and Arts of Baden-Wuerttemberg, Germany.

The research question and methods for this paper were pre-registered on aspredicted.org (see https://aspredicted.org/DBC_WFK). This paper, however, only includes research question 1 of the pre-registration. Research question 2 of the pre-registration will be reported elsewhere. Pseudonymised data and analysis scripts are openly available at the Open Science Framework (see https://osf.io/s87mu/?view_only=None).

## Intervention

Randomization of participants was performed by block randomization using sealed envelopes with blocks of ten to allocate the participants 1:1 either into the control group with standard care (CG) or the experimental group with the additional study intervention (EG). Each woman assigned to the EG was (1) supervised by a “pregnancy companion” from our team of specially trained midwives and medical assistants, (2) received approximately 6 one-on-one virtual health-coaching sessions and (3) had access to the Buddy Healthcare app (Buddy Healthcare HQ, Helsinki, Finland) to share educational materials, facilitate communication, and collect data on physical activity (average steps per day delivered once a week).

The video calls between the participants of the EG and the pregnancy companion took place with a minimum of every 4 weeks and were delivered via Doctolib App (Doctolib GmbH, Berlin, Germany). Pregnancy companions were extensively trained to use motivational interviewing strategies. The duration of the video calls ranged from 10 to 60 min, depending on the women’s needs. The structured health-coaching sessions included giving advice on a healthy lifestyle such as physical activity and a healthy nutrition but also provided the opportunity to talk about pregnancy symptoms, worries and fears, partnership, etc. Pregnancy-related information adapted to the gestational age was accessible through the Buddy Healthcare app for every woman in the EG. Questionnaires and video calls were employed to evaluate the individual circumstances of each woman in the EG. Upon identifying a need for further counseling, relevant information pertaining to diverse subjects, such as diet, addiction, financial situation, or single parenting, was provided. If required, the “pregnancy companion” also facilitated the process of reaching out to counseling centers. Additional individual information was delivered via the app to reinforce content discussed during the video calls. Between the appointments with the pregnancy companion, participants from the experimental group had the opportunity to contact the healthy birth staff via chat if needed.

Two of the primary goals of the intervention were to increase steps per day and to avoid excessive weight gain.

### Standard care

Women of both groups received a fitness tracker wrist (Huawei 6) to self-monitor physical activity and agreed to wear the device during the day for the whole duration of their pregnancy.

The Buddy Healthcare app was also used to administer questionnaires about demographic information by the time of study enrollment and about participant satisfaction after conclusion of the program. Women with standard care only received a different version of the app as they did not receive pregnancy-related information, nor did they have the possibility to use the chat function. After delivery, every participant was asked to submit the final data of her maternity log and medical record from the hospital via app.

Furthermore, every participant (CG and EG) attended obstetric standard care with regular scheduled visits to their obstetricians and midwives according to the maternity guidelines in Germany [22]. These visits took place every 4 weeks until week 32 and then every 2 weeks until delivery if the course of the pregnancy was uneventful and the risk profile of gestation was low.

### Participants

**Fig. 1:**
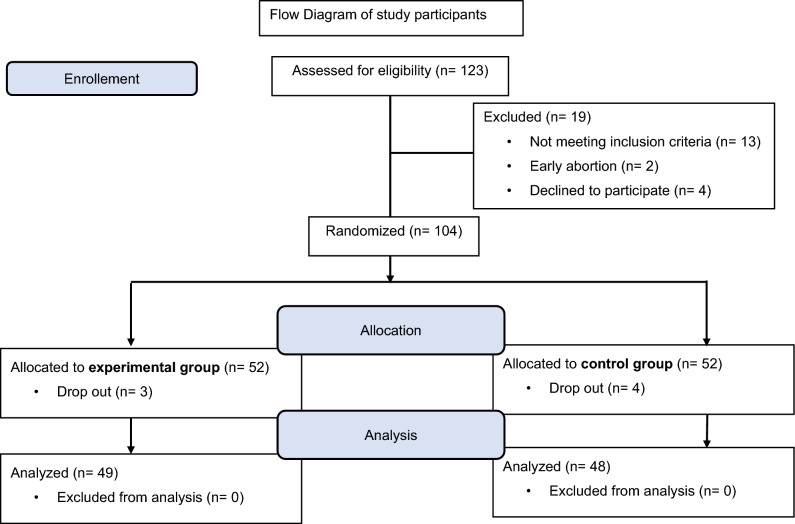
Flowchart of study participants

Participants were recruited during their first trimester using referrals from prenatal care providers, information tables and social media between January 02, 2022 and August 08, 2022. Using these methods, we recruited a convenience sample of women between 18 and 40 years of age, who owned a smartphone and were able to comprehend German. Women were excluded when the pregnancy was already more than 20 gestational weeks by the time of the first contact. Women with contraindication to exercise or physical limitations that prevented exercise were not included in the analysis. All participants provided written informed consent, including that research personnel may abstract prenatal, birth, and postpartum clinical data from their electronic health record. Participants whose data could be collected up to the end of January 2023 were included. Figure 1 shows the flow of participants through the study. Dropout rate was 8% (CG) resp. 6% (EG). Data from 97 women were analyzed.

### Measures

On the day of study enrollment, every woman (CG and EG) received a fitness tracker wrist. Once a week, the participants had to type the average steps per day from the last week into the Buddy Healthcare app. If data transfer did not occur, participants were reminded via the app.

Our primary outcome to assess the efficacy of the intervention for improvement of physical activity was change in steps per day. The average steps per day during the first three weeks of the study were taken as the baseline steps for each patient. Further values entered until week 26 were taken to calculate the mean by the end of the second trimester. Values from week 27 until week 38 were taken to calculate the mean by the end of the third trimester. Absolute step values per day between both groups were compared.

We additionally explored the proportion of participants who suffered from excessive gestational weight gain (EGWG). Gestational weight gain (GWG) was calculated by subtracting the weight before pregnancy from the most recent weight taken before delivery. EGWG was defined as GWG exceeding the recommended GWG for each pre-pregnancy BMI category by gestational age at delivery on the basis of the 2009 Institute of Medicine (IOM) guidelines [23].

Demographic information was collected with a baseline questionnaire by the time of the study enrollment. Medical and reproductive history as well as data from the current pregnancy was assessed after delivery and taken from participant’s medical records and maternity logs. BMI and weight change during pregnancy were calculated using data from the maternity log.

### Statistical analysis

Analyses were conducted using Jamovi, version 2.2.5 and R, version 4.2.1. To compare group means of absolute steps per day at the end of the intervention, i.e., the third trimenon of pregnancy, an independent samples *t* test (two-sided) was employed. As a robustness check, we additionally conducted a Wilcoxon rank/Mann–Whitney *U* test, because the normality assumption was violated. A *p* value of less than 0.05 indicated a significant effect. Between-group effect sizes were calculated by the standardized group mean difference bias-corrected for small sample sizes (i.e., Hedges' *g*; Hedges and Olkin [24]). In an additional exploratory analysis, efficacy was determined using a repeated-measures ANOVA to assess changes in steps per day across three measurement points (baseline as well as the second and third trimester). This within-subjects design offers the advantage that each participant serves as her own control, effectively eliminating variance attributed to individual differences. Chi-square tests were conducted to assess differences in excessive weight gain and an independent samples *t* test was used to assess differences in baseline characteristics between groups.

Post hoc power was estimated via G*Power 3.1 [25]. Post hoc power for our main analysis, i.e., the independent samples *t* test (two-sided) between the CG (*n* = 48) and the EG (*n* = 49) and an alpha error of 0.05, was 1−*β* = 0.97 to detect a large effect, 1−*β* = 0.68 to detect a medium effect, and 1−*β* = 0.16 to detect a small effect.

## Results

Baseline characteristics for both groups are listed in Table 1. Participants in the CG were more likely to have a history of abortion than in the EG (*p* = 0.005). No other differences in baseline characteristics were detected.

**Table 1:** Maternal characteristics

	Intervention *n* = 49	Control *n* = 48	Total *n* = 97
Age, mean years $\pm$ SD	32 (4)	32 (4)	32 (4)
Education, *n* (%)			
University degree	30 (61)	26 (54)	56 (58)
Employment			
Full time	27 (55)	27 (56)	54 (56)
Part time	18 (37)	14 (29)	32 (33)
Homemaker	2 (4)	4 (8)	6 (6)
Unemployed	2 (4)	3 (6)	5 (10)
Prepregnancy BMI, mean kg/m^2^ $\pm$ SD, *n* = 95	25 (5)	25 (4)	25 (4)
Prepregnancy BMI category, n (%), *n* = 95			
Underweight (< 18.5 kg/m^2^)	0 (0)	2 (4)	2 (2)
Normal (18.5–24.9 kg/m^2^)	30 (63)	25 (53)	55 (58)
Overweight (25–29.9 kg/m^2^)	10 (21)	15 (32)	25 (26)
Obese ($\ge$ 30 kg/m^2^)	8 (17)	5 (11)	13 (14)
Parity, *n* (%)			
Primipara	23 (47)	25 (52)	48 (50)
Pregnancy history, *n* (%)			
History of gestational hypertension	1 (2)	0 (0)	1 (1)
History of pre-eclampsia	5 (10)	0 (0)	5 (5)
History of gestational diabetes	1 (2)	1 (2)	2 (2)
History of preterm birth	4 (8)	1 (2)	5 (5)
History of abortion	11 (22)	24 (50)	35 (36)
Infertility treatment, *n* (%), *n* = 95 (%)	4 (8)	6 (13)	10 (11)

**Fig. 2:**
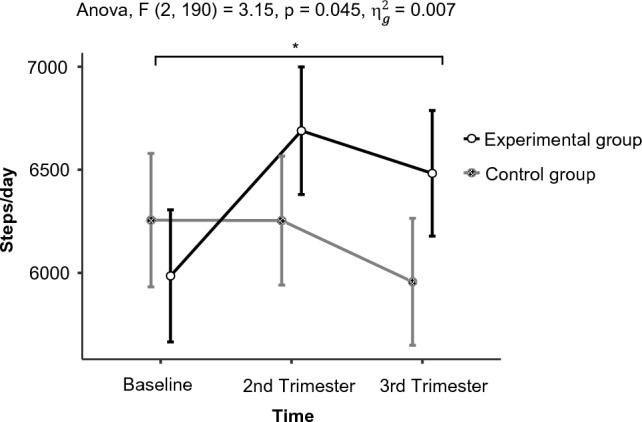
Changes in step value over time in mean steps/day

Primary outcome change in steps per day and exploratory outcome change in weight are presented in Table 2. The absolute step count did not differ between the two groups at the third trimenon (*p* = 0.227). Thus, the small effect size of *g* = 0.24 did not reach significance either (95% CI [0.16; 0.65]). The repeated-measures ANOVA (see Fig. 2) revealed no significant main effects of either factor, i.e., time or treatment group, but a significant interaction effect, more specifically, a cross-over effect (*p* = 0.045). That is, the effect of time on the amount of steps was opposite depending on the treatment group, meaning that women of the treatment group started with a lower step count than in the control group, but have an overall increase of steps per day toward the end. Mothers of the control group, however, started with a higher step count on average, but showed an overall decrease toward the end of pregnancy.

**Table 2:** Physical activity and maternal weight gain

	Control group *n* = 48	Intervention group *n* = 49	*p* value
Physical activity, mean steps/day $\pm$ SD			
Baseline	6256 $\pm$ 2429	5986 $\pm$ 2038	0.554
2nd trimester	6254 $\pm$ 2143	6689 $\pm$ 2190	0.325
3rd trimester	5957 $\pm$ 2242	6483 $\pm$ 2017	0.227
Change from baseline, mean steps/day $\pm$ SD			
to 2nd trimester	− 2 $\pm$ 1844	704 $\pm$ 1764	0.057
to 3rd trimester	− 300 $\pm$ 1902	497 $\pm$ 1816	**0.038**

	Control Group *n* = 44	Intervention Group *n* = 43	*p* value
Gestational weight gain, mean kg $\pm$ SD	14.3 (4.8)	12.8 (3.9)	0.126
IOM* gestational weight gain guidelines, *n* (%)			
Below	8 (19)	10 (22)	0.716
Within	11 (26)	21 (47)	**0.048**
Above	23 (55)	14 (31)	**0.026**

*Institute of Medicine

Bold indicates values that are significant (*p* < 0.05)

The proportion of participants who met the IOM recommendation for total weight gain during pregnancy was significantly higher in EG than in CG (*p* = 0.048), and the ratio of women that gained excessively was higher in the CG (*p* = 0.026). There was no significant difference in absolute weight gain in either group, although, on a descriptive level, participants in the intervention group gained less weight on average compared with the control group ($-$ 1.43 kg; 95%CI $[-$ 0.41, 3.28]; *p* = 0.126).

Additional exploratory outcomes of interest analyzed in the study are presented in Table 3 and did not show any differences among maternal or infant outcomes.

**Table 3:** Pregnancy outcomes

	Intervention (*n* = 49)	Control (*n* = 48)	*p* value
Gestational age at delivery, *n* = 97, mean weeks $\pm$ SD	40 (1.5)	40 (1.3)	0.787
Adverse pregnancy outcomes, *n* = 96, *n* (%)			
Preterm delivery	2 (4)	2 (4)	0.983
Gestational diabetes	7 (14)	5 (11)	0.594
Gestational hypertension	1 (2)	0 (0)	0.330
Hypertensive pregnancy disorder	1 (2)	1 (2)	0.977
Intrauterine growth restriction	0 (0)	2 (4)	0.148
All adverse pregnancy outcomes	11 (22)	10 (21)	0.891
Mode of delivery, *n* = 97, *n* (%)			
Vaginal delivery	30 (61)	34 (71)	0.323
Cesarean section	19 (39)	14 (29)	0.323
Infant outcomes			
Male, *n* (%)	25 (51)	23 (48)	0.763
Birthweight, mean grams $\pm$ SD	3451 (544)	3473 (537)	0.843
Birth length, mean cm $\pm$ SD	52 (3.1)	52 (2.5)	0.689

Conducting sensitivity analyses, that is, excluding outliers, did not majorly change our results.

## Discussion

The aim of the present study was to examine the impact of a personal multifaceted intervention on physical activity and excessive weight gain in women of all BMI categories. We combined integrated personal support by a pregnancy companion and an interprofessional team with the use of digital technology to achieve targeted antenatal care. The mobile app facilitates communication and provides individualized phase-specific information on pregnancy, healthy lifestyle, mental health, partnership, and social support options. Wearable activity trackers provide reliable feedback on physical performance.

The pre-registered hypothesis, presuming a significant difference in absolute step values between the two groups in the third trimester, could not be confirmed. Nor could a significant difference in our exploratory measure, i.e., the absolute weight gain, be detected.

However, while the step count in CG decreased by an average of 300 steps per day from baseline to the third trimester, the intervention group showed an increase of 497 steps per day. These descriptive findings could be corroborated by a significant cross-over effect in an exploratory repeated-measures ANOVA, which takes the within-in person effects into account. This result suggests that the integrated support of our program leads to a higher individual level of physical activity during the course of pregnancy. Since physical activity levels typically decrease during the final weeks of pregnancy, even a small increase in activity or maintaining one’s activity level can have important benefits [18].

While no difference in absolute weight gain was detected, the proportion of women exceeding the IOM guidelines for gestational weight gain was significantly lower in EG (*p* = 0.026). These results are in line with a randomized-controlled trial with 394 overweight or obese pregnant women from Ferrara et al., evaluating the effectiveness of a telehealth lifestyle intervention. The intervention included 13 weekly individual sessions delivered by dietitians and led to a significant reduction of the proportion of women exceeding the IOM guidelines [26]. In contrast, an online behavioral intervention by Olsen et al. with 1689 women did not show a difference in EGWG. The intervention in this randomized-controlled trial included behavior change tools such as a weight gain tracker, a diet and a physical activity goal-setting and a self-monitoring tool but did not provide any personal support [27].

Our result of the exploratory analysis is consistent with a recent systematic review and meta-analysis of 1934 pregnant women and 18 randomized-controlled trials comprising all kinds of interventions during pregnancy and only trials in which data were measured by a technical device. Based on their findings, the authors resolved that women randomized to a physical activity intervention group achieved 435 more steps per day at the end of the pregnancy (95%CI [− 0.5, 870.6], *p* = 0.05). The average steps per day at baseline for intervention and comparator groups in the meta-analysis (6704 and 6327 steps, respectively) were comparable to our findings (6256 and 5986 steps, respectively) [28].

Atkinson et al. performed a randomized-controlled trial with 241 women in Canada to assess the effect of weekly in-person counseling focusing on dietary goals and walking-based exercise programs. The authors observed no significant differences in step count or rates of excessive weight gain between both groups [29], corroborating the conjecture how difficult it apparently is to achieve a behavioral change during pregnancy or at least maintain the level of activity. A randomized-controlled trial by Kennelly et al. involved 565 women from Ireland and established that an intervention of antenatal behavior change supported by smartphone application technology led to higher levels of physical activity in the third trimester. In distinction to our work, only overweight and obese women were included, the behavior change intervention was a single face-to-face education session at the first study visit, and levels of physical activity were self-reported [30].

Healthy lifestyle counseling appears to be insufficient in routine antenatal care and should focus on the individual needs of each woman and her family to be of sustainable success [31–33]. This complex care can be provided through individual support from a team of experienced professionals. However, to fully leverage the advantages of digital technologies, this support should also incorporate digitals tools and platforms.

Limitations of the study relate to the generalizability of the findings. Our study population was predominantly educated with most of the women holding a university degree. To improve the applicability of the study’s results, future research should include women from low socioeconomic backgrounds. It seems likely that this type of intervention would have a more significant impact on a population with a greater need for these resources. A further limitation is the low power of our study to detect small and medium effects.
